# NLR–TyG index and prevalent chronic kidney disease in hypertension: a cross-population study

**DOI:** 10.3389/fnut.2026.1917577

**Published:** 2026-10-19

**Authors:** Honglin Li, Chuanwei Zhao, Mingpeng Zhao, Xinhua Wu

**Affiliations:** 1 Department of General Practice, People's Hospital of Tengchong City, Tengchong, Yunnan, China; 2 Department of Cardiology, The Second People’s Hospital of Baoshan City Yunnan Province, Baoshan, Yunnan, China; 3 Department of Cardiology, The First Affiliated Hospital of Dali University, Dali, Yunnan, China; 4 College of Health Science and Technology, West Yunnan University of Applied Sciences, Dali, Yunnan, China

**Keywords:** chronic kidney disease, hypertension, inflammation, insulin resistance, neutrophil-to-lymphocyte ratio, NHANES, NLR–TyG index, triglyceride–glucose index

## Abstract

**Background:**

Inflammation and metabolic dysfunction are interrelated in chronic kidney disease (CKD). The NLR–TyG index (NTI), calculated from the neutrophil-to-lymphocyte ratio (NLR) and triglyceride–glucose (TyG) index, summarizes concurrent inflammatory and metabolic burden. We examined its association with prevalent CKD in hypertensive adults from a Chinese cardiology inpatient population and the US National Health and Nutrition Examination Survey (NHANES).

**Methods:**

This cross-sectional study included hypertensive adults from the Department of Cardiology, First Affiliated Hospital of Dali University, and NHANES. NTI was analyzed continuously and by cohort-specific quartiles. Multivariable logistic regression and restricted cubic splines assessed associations. Subgroup, sensitivity, and incremental discrimination analyses were also performed.

**Results:**

The analysis included 8,358 hospital participants (1,800 with CKD) and 9,182 NHANES participants (2,751 with CKD). In fully adjusted models, each 1-unit increase in NTI was associated with higher odds of CKD in the hospital population [odds ratio (OR), 1.012; 95% confidence interval (CI), 1.010–1.014] and NHANES (OR, 1.021; 95% CI, 1.013–1.029; both *p* < 0.001). Compared with the lowest NTI quartile, the highest quartile was associated with higher CKD odds in the hospital population (OR, 2.85; 95% CI, 2.38–3.40) and NHANES (OR, 1.93; 95% CI, 1.56–2.38; both P for trend <0.001). Significant nonlinearity was detected only in the hospital population. Associations persisted among participants with known non-use of lipid-lowering therapy. Adding NTI increased the C statistic from 0.769 to 0.778 in the hospital population and from 0.772 to 0.778 in NHANES; discrimination was similar when NLR and TyG were entered separately.

**Conclusion:**

Higher NTI was independently associated with prevalent CKD in both hypertensive populations and provided modest incremental discrimination. NTI may offer a simple summary of inflammatory–metabolic burden, but prospective prediction and clinical utility require further validation.

## 1 Introduction

Chronic kidney disease (CKD) is a major public health problem, particularly among adults with hypertension. National surveillance in China and the United States has shown a substantial burden of CKD in populations with hypertension, underscoring the close relationship between blood-pressure disorders and impaired kidney function (1, 2). Contemporary cardiovascular-kidney-metabolic frameworks further emphasize that hypertension, metabolic dysfunction, cardiovascular disease, and CKD are interrelated rather than isolated conditions (3). Systemic inflammation and insulin resistance are two biological processes that may contribute to this overlap. The neutrophil-to-lymphocyte ratio (NLR), derived from a routine complete blood count, reflects the balance between neutrophil-predominant innate immune activation and lymphocyte-related adaptive immune regulation. Activated neutrophils participate in inflammatory signaling through reactive oxygen species, proteolytic enzymes, cytokine release, and neutrophil extracellular traps, whereas changes in lymphocyte counts may accompany physiological stress and altered immune homeostasis; accordingly, NLR is widely used as an accessible marker of systemic inflammatory-immune balance rather than as a direct measure of inflammation itself (4). Recent prospective evidence has also linked higher NLR with incident CKD (5). The triglyceride-glucose (TyG) index represents a complementary biological domain. Originally proposed as a surrogate marker of insulin resistance using fasting triglyceride and glucose concentrations (6), TyG has shown moderate diagnostic utility for insulin resistance in systematic evaluation (7). Insulin resistance and glucose-lipid dysregulation may adversely affect the kidney through endothelial dysfunction, oxidative stress, altered renal hemodynamics, and abnormal lipid handling. Consistent with these mechanisms, higher TyG has been associated with incident or worsening renal dysfunction in longitudinal studies (8). Thus, NLR and TyG provide complementary information on inflammatory and metabolic states that are both relevant to renal vulnerability.

The coexistence of inflammatory and metabolic abnormalities provides a rationale for evaluating the two domains jointly, but it does not imply that any particular mathematical combination is biologically synergistic or statistically optimal. In the present study, the NLR–TyG index (NTI) was prespecified as the product of NLR and TyG to provide a simple, unweighted representation of concurrent inflammatory and metabolic burden. Because both components are positive continuous measures, their product increases when either component rises and is particularly elevated when both are simultaneously high. Importantly, NTI was not derived from outcome-dependent regression coefficients or data-driven weighting, and the product form should not be interpreted as proof of a multiplicative biological interaction. This distinction is relevant because emerging studies have begun to evaluate combined inflammatory-metabolic indicators, including the joint use of TyG and NLR in the general US population (9), the concurrent roles of NLR and TyG in CKD risk stratification among patients with coronary artery disease (10), and other composite inflammatory-metabolic indices such as the C-reactive protein-TyG index in CKD (11). These studies support growing interest in integrated inflammatory-metabolic phenotyping while also indicating that the clinical meaning of any composite depends on its population, outcome, and analytic formulation. To avoid assuming that the product formulation is intrinsically superior, our analysis also compared NTI with its individual components and with alternative parameterizations.

Accordingly, we evaluated the association between NTI and prevalent CKD in two clinically and demographically distinct populations of adults with hypertension: a Chinese cardiology inpatient cohort and hypertensive participants from the nationally representative US National Health and Nutrition Examination Survey (NHANES). The study was designed to characterize NTI as both a continuous measure and cohort-specific quartiles, examine its dose–response association with prevalent CKD, assess the consistency of associations across clinically relevant subgroups and sensitivity analyses, and quantify its incremental discriminative information beyond conventional covariates. We also compared NTI with NLR and TyG considered separately and examined alternative parameterizations, thereby addressing whether a simple multiplicative composite provides reproducible epidemiological information without presuming that it constitutes a validated clinical prediction score. This cross-population approach was intended to clarify the potential value and the interpretative limits of NTI as a parsimonious marker of concurrent inflammatory and metabolic burden in adults with hypertension.

## 2 Materials and methods

### 2.1 Study design and data sources

This cross-sectional analysis used two independent data sources: a retrospective hospital-based cohort from the Department of Cardiology, First Affiliated Hospital of Dali University, Yunnan Province, China, and the US NHANES. The hospital cohort comprised routinely collected inpatient data from March 2022 through June 2025. Clinical diagnoses, medication information, demographic characteristics, and laboratory measurements were obtained from the hospital electronic medical record and laboratory information systems. The NHANES component included survey periods from 1999 to 2000 through the 2017–March 2020 pre-pandemic release. NHANES uses a complex, multistage probability sampling design to obtain nationally representative health and nutrition information for the US civilian, non-institutionalized population (12, 13).

The hospital-based study was approved by the Ethics Committee of the Second People’s Hospital of Baoshan City, Yunnan Province, China (Approval No. 2025–032 (Other)-001), which covered the retrospective use of de-identified data from the First Affiliated Hospital of Dali University analyzed in this study. The study was conducted in accordance with the Declaration of Helsinki, and the requirement for written informed consent was waived because only de-identified retrospective data were used. NHANES protocols were approved by the National Center for Health Statistics Ethics Review Board, and written informed consent was obtained from all participants (14).

### 2.2 Study population

For the hospital cohort, 18,341 source records were screened. Hypertension was identified from inpatient discharge-diagnosis fields using International Classification of Diseases (ICD), Tenth Revision (ICD-10) codes I10 [essential (primary) hypertension], I11 (hypertensive heart disease), I12 (hypertensive renal disease), I13 (hypertensive heart and renal disease), or I15 (secondary hypertension). Use of ICD-coded electronic health-record and administrative data for hypertension ascertainment is well established in epidemiological research, including Chinese electronic health-record studies (15, 16). We excluded 8 exact duplicate records, 138 records from participants aged <20 years or with missing age, and 7,876 records without hypertension, leaving 10,319 records. We then excluded 1,552 records without valid positive values for the components required to calculate the NTI, 389 records in which CKD status could not be ascertained, and 20 participants receiving dialysis. The final hospital-based analytic sample therefore comprised 8,358 participants.

Across the selected NHANES periods, 107,622 participants were screened. Hypertension was defined by any of the following: mean systolic blood pressure (SBP) ≥ 140 mmHg, mean diastolic blood pressure (DBP) ≥ 90 mmHg, self-reported physician or health-professional diagnosis of hypertension, or current use of prescribed antihypertensive medication. Mean SBP and DBP were calculated from the available standardized examination readings. This combined definition is consistent with established NHANES hypertension surveillance approaches integrating measured blood pressure, self-reported diagnosis, and treatment status (17). We excluded 48,878 participants aged <20 years or with missing age and 34,184 without hypertension, leaving 24,560 participants. We subsequently excluded 13,977 participants without valid positive NTI components, 147 with unascertainable CKD status, 64 receiving dialysis, 716 with zero or missing official fasting-subsample weights, and 474 with missing covariates required for the multivariable models. The final NHANES analytic sample comprised 9,182 participants. Participant selection is shown in Figure 1.

**Figure 1:**
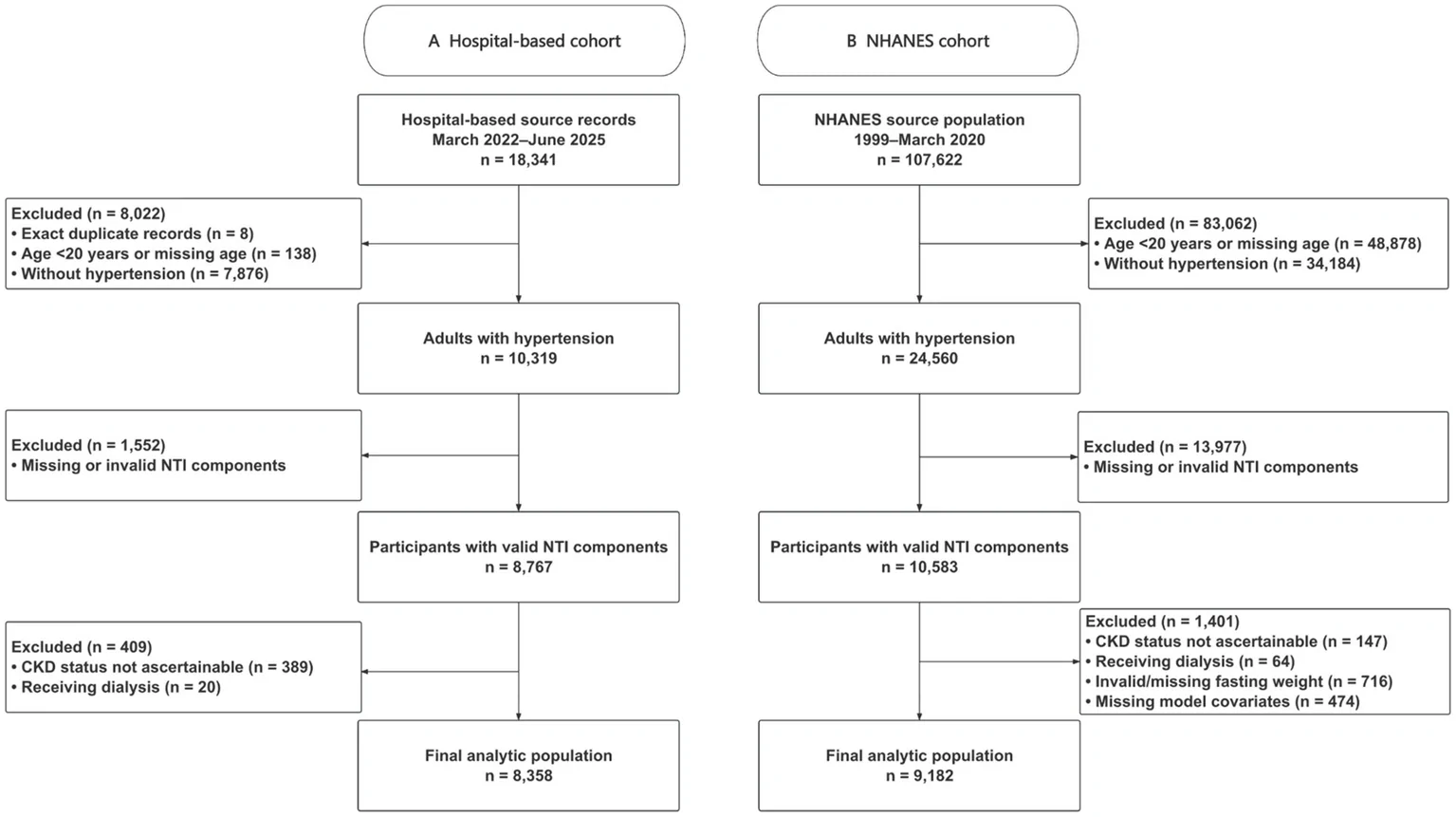
Participant selection in the hospital-based and NHANES populations. **(A)** Department of Cardiology, First Affiliated Hospital of Dali University cohort, March 2022–June 2025. **(B)** National Health and Nutrition Examination Survey (NHANES), 1999–March 2020. Main boxes show records retained after each sequential step and side boxes show exclusions. NTI, neutrophil-to-lymphocyte ratio × triglyceride–glucose index; CKD, chronic kidney disease.

### 2.3 Exposure assessment

The primary exposure was the NTI, which combines an inflammatory marker with a metabolic marker. The NLR was calculated as the absolute neutrophil count divided by the absolute lymphocyte count. The triglyceride–glucose (TyG) index was calculated as ln[triglycerides (mg/dL) × fasting glucose (mg/dL)/2] (8). For the hospital cohort, triglyceride and fasting glucose concentrations recorded in mmol/L were converted to mg/dL before calculation (triglycerides × 88.57 and glucose × 18). NTI was then calculated as NLR × TyG. NTI was prespecified as a parsimonious composite measure intended to summarize concurrent inflammatory and metabolic burden. No outcome-derived weighting, coefficient optimization, or data-driven cutoff selection was used to construct the index. The product form was not interpreted as evidence of a biological or statistical interaction between NLR and TyG.

### 2.4 Outcome ascertainment

The study outcome was prevalent CKD. In the hospital cohort, CKD was defined as either a recorded CKD diagnosis in the medical record or an estimated glomerular filtration rate (eGFR) < 60 mL/min/1.73 m^2^. Hospital eGFR was recalculated using the 2009 Chronic Kidney Disease Epidemiology Collaboration (CKD-EPI) creatinine equation (18). Urine albumin-to-creatinine ratio (UACR) was not available for the hospital outcome. In NHANES, CKD was defined as eGFR <60 mL/min/1.73 m^2^ or UACR ≥30 mg/g, consistent with accepted CKD criteria (19). eGFR values were obtained from the harmonized source dataset, and UACR was derived from the official urine albumin and urine creatinine components. Participants receiving dialysis were excluded from both cohorts. Because UACR was unavailable in the hospital dataset, CKD ascertainment was not identical across the two populations.

### 2.5 Covariates

Covariates were selected *a priori* according to established CKD risk factors, biological plausibility, and prior literature rather than on the basis of univariable statistical significance (19). The prespecified adjustment set included age, sex, race/ethnicity, education, marital status, smoking status, drinking status, body mass index (BMI), SBP, diabetes mellitus, and heart failure. For both cohorts, sex was classified as female or male; educational attainment as below high school or high school and above; marital status as married or other; smoking status as never, former, or current; and drinking status as current alcohol use or non-current alcohol use. Ethnicity/race was classified separately because of differences between data sources: participants in the hospital cohort were categorized as Han or other ethnic groups, whereas NHANES participants were categorized as non-Hispanic White or other race/ethnicity. BMI was calculated as body weight in kilograms divided by height in meters squared (kg/m^2^), consistent with the World Health Organization definition (20). BMI was modeled continuously in the primary multivariable analyses and dichotomized as <25 versus ≥25 kg/m^2^ only for the prespecified subgroup analysis.

In the hospital cohort, diabetes mellitus was identified from discharge-diagnosis fields using ICD-10 codes E10 (type 1 diabetes mellitus), E11 (type 2 diabetes mellitus), E12 (malnutrition-related diabetes mellitus), E13 (other specified diabetes mellitus), or E14 (unspecified diabetes mellitus). The E10–E14 ICD-10 code family is widely used for diabetes ascertainment in administrative and hospital data and has demonstrated good validity against clinical records (21). In NHANES, diabetes mellitus was defined by any of the following: fasting plasma glucose ≥126 mg/dL, hemoglobin A1c (HbA1c) ≥ 6.5%, self-reported physician or health-professional diagnosis of diabetes other than gestational diabetes, or current use of insulin or oral glucose-lowering medication. These criteria are consistent with established diagnostic thresholds and commonly used NHANES diabetes definitions (22, 23). Heart failure was identified from physician diagnosis or structured medical-record documentation as represented in each source dataset. Lipid-lowering therapy was defined as medication use before admission in the hospital cohort and current medication use in NHANES and was used in the prespecified sensitivity analysis. Laboratory variables used for descriptive characterization included triglycerides, total cholesterol, high-density lipoprotein cholesterol, low-density lipoprotein cholesterol, serum creatinine, fasting plasma glucose, neutrophil count, lymphocyte count, NLR, TyG, and NTI.

### 2.6 Statistical analysis

Continuous-variable distributions were assessed separately in each cohort using histograms, quantile–quantile plots, skewness, and the Shapiro–Wilk test. Because the Shapiro–Wilk test is highly sensitive in large samples, it was interpreted together with graphical and distribution-shape assessments; when more than 5,000 nonmissing observations were available, the test was performed on a fixed random sample of 5,000 observations. The evaluated continuous variables showed departures from normality and were therefore summarized as medians with interquartile ranges (IQRs). In the hospital cohort, continuous variables were compared using Wilcoxon rank-sum tests and categorical variables using Pearson chi-square tests. In NHANES, continuous variables were summarized using survey-weighted medians and IQRs and compared using survey-weighted Wilcoxon rank-sum tests; categorical variables were presented as unweighted counts with survey-weighted percentages and compared using Rao–Scott chi-square tests.

NTI was analyzed both continuously and categorically. The continuous analysis used the raw NTI scale and reported the odds ratio (OR) per 1-unit increase. Cohort-specific quartiles were defined from the empirical NTI distribution within each analytic cohort. The quartile cutoffs were 14.000, 19.622, and 28.809 in the hospital cohort and 13.118, 17.893, and 24.360 in NHANES. Cohort-specific quartiles were used because the NTI distributions differed between the two populations and the purpose was to compare relative exposure categories within each cohort rather than to impose a common clinical threshold. Quartile boundaries were derived from the unweighted analytic distributions, whereas all NHANES association models incorporated the complex survey design. NTI distributional characteristics and quartile boundaries are presented in Supplementary Table S1 and Supplementary Figure S1.

Associations between NTI and prevalent CKD were estimated using logistic regression in the hospital cohort and survey-weighted logistic regression in NHANES, with results reported as ORs and 95% confidence intervals (CIs). The crude model included NTI alone. Model 1 adjusted for age, sex, race/ethnicity, education, and marital status. Model 2 additionally adjusted for smoking, drinking, BMI, SBP, diabetes, and heart failure. NTI quartiles were entered as categorical terms with Q1 as the reference, and P for trend was evaluated across increasing quartiles.

Restricted cubic spline models were used to characterize the dose–response association between NTI and prevalent CKD (24). Knots were placed at the cohort-specific 10th, 50th, and 90th percentiles of NTI, and the cohort-specific median was used as the reference value. Overall association was evaluated by jointly testing the spline terms, and nonlinearity was assessed from the nonlinear spline component. Hospital spline models used conventional logistic-regression inference, whereas NHANES spline models used survey-adjusted inference accounting for weights, strata, and primary sampling units.

Prespecified subgroup analyses were performed according to age (<60 vs. ≥ 60 years), sex, race/ethnicity, smoking, drinking, diabetes, and BMI (<25 vs. ≥ 25 kg/m^2^). Stratum-specific associations were estimated using the Model 2 adjustment framework, omitting the stratification variable within the corresponding stratum. Effect modification was evaluated using formal NTI-by-subgroup interaction terms. Interaction tests used nested likelihood-ratio tests in the hospital cohort and survey-adjusted joint Wald *F* tests in NHANES. Sensitivity analyses were restricted to participants with known non-use of lipid-lowering therapy, excluding both users and those with unknown medication status. The primary covariate adjustment sets and cohort-specific NTI quartile cutoffs were retained.

Incremental discriminative performance was assessed relative to the basic Model 2 covariates. We compared the basic model with models additionally containing TyG, NLR, NTI, or NLR and TyG entered as separate terms. Discrimination was quantified using the C statistic and change in C statistic, continuous net reclassification improvement (NRI), and integrated discrimination improvement (IDI) (25). Ninety-five percent CIs for these metrics were obtained from 250 bootstrap replicates. Added terms were evaluated using likelihood-ratio tests in the hospital cohort and survey-adjusted Wald *F* tests in NHANES. Additional analyses evaluated standardized log-transformed NTI and a formal NLR × TyG interaction term.

For NHANES, analyses followed National Center for Health Statistics guidance for complex survey data (12, 13). Official fasting-subsample weights were used and combined across survey periods in proportion to the duration represented by each period; the 2017–March 2020 pre-pandemic release was treated as a 3.2-year period. Survey strata and primary sampling units were incorporated in all weighted models. Participants with zero or missing fasting-subsample weights were excluded. After categorical harmonization, participants with residual missing BMI or SBP were excluded during final NHANES cohort construction; no multiple imputation was performed. Analyses were performed using R version 4.3.2. All tests were two-sided, and *p* < 0.05 was considered statistically significant.

## 3 Results

### 3.1 Study population and baseline characteristics

The final analysis included 8,358 participants in the hospital cohort, of whom 1,800 had prevalent CKD, and 9,182 participants in NHANES, of whom 2,751 had prevalent CKD (Figure 1). The median age was 63 years in the hospital cohort, and the survey-weighted median age was 57 years in NHANES. Women accounted for 41.58% of the hospital cohort and a survey-weighted 50.65% of the NHANES population. Baseline characteristics according to CKD status are presented in Tables 1, 2. Participants with CKD were older in both populations (hospital: 71 vs. 61 years; NHANES: 69 vs. 55 years; both *p* < 0.001) and had a greater burden of diabetes and heart failure. They also had higher neutrophil counts, NLR, and NTI. In the hospital cohort, median NTI was 25.91 (IQR 18.02–40.80) in participants with CKD and 18.31 (13.38–26.17) in those without CKD. In NHANES, the corresponding survey-weighted medians were 21.01 (15.48–28.43) and 17.64 (13.30–23.70), respectively (both *p* < 0.001).

**Table 1:** Baseline characteristics of hypertensive participants according to CKD status in the hospital-based population.

Variable	Total (*N* = 8,358)	Non-CKD (*n* = 6,558)	CKD (*n* = 1,800)	*p* value
Age, years	63.00 (54.00–72.00)	61.00 (53.00–70.00)	71.00 (62.00–78.00)	<0.001
BMI, kg/m^2^	24.23 (23.18–25.39)	24.38 (23.36–25.49)	23.67 (22.65–24.89)	<0.001
TG, mmol/L	1.54 (1.10–2.26)	1.58 (1.12–2.32)	1.39 (1.02–2.03)	<0.001
TC, mmol/L	4.33 (3.58–5.12)	4.40 (3.63–5.17)	4.09 (3.38–4.95)	<0.001
HDL-C, mmol/L	1.10 (0.91–1.32)	1.10 (0.92–1.32)	1.09 (0.90–1.32)	0.175
LDL-C, mmol/L	2.22 (1.67–2.82)	2.27 (1.71–2.85)	2.05 (1.58–2.73)	<0.001
Serum creatinine, μmol/L	78.00 (64.00–96.00)	72.00 (61.00–84.00)	123.00 (104.00–157.25)	<0.001
Lymphocytes, ×10^9^/L	1.72 (1.31–2.18)	1.79 (1.39–2.24)	1.42 (1.04–1.88)	<0.001
Neutrophils, ×10^9^/L	3.81 (2.96–5.02)	3.72 (2.91–4.84)	4.27 (3.20–5.69)	<0.001
SBP, mmHg	136.00 (121.00–151.00)	137.00 (123.00–152.00)	131.00 (115.00–147.00)	<0.001
DBP, mmHg	82.00 (72.00–93.00)	83.00 (74.00–94.00)	76.69 (67.00–89.00)	<0.001
FPG, mmol/L	5.32 (4.79–6.29)	5.30 (4.81–6.17)	5.44 (4.71–6.75)	0.009
TyG index	8.83 (8.43–9.30)	8.85 (8.45–9.32)	8.76 (8.37–9.25)	<0.001
NLR	2.20 (1.58–3.24)	2.06 (1.51–2.95)	2.96 (2.02–4.62)	<0.001
NTI	19.62 (14.00–28.81)	18.31 (13.38–26.17)	25.91 (18.02–40.80)	<0.001
Sex				0.147
Female	3,475 (41.58%)	2,754 (41.99%)	721 (40.06%)	
Male	4,883 (58.42%)	3,804 (58.01%)	1,079 (59.94%)	
Ethnicity				0.489
Han	4,674 (55.92%)	3,654 (55.72%)	1,020 (56.67%)	
Other	3,684 (44.08%)	2,904 (44.28%)	780 (43.33%)	
Education				<0.001
Below high school	6,448 (77.15%)	4,974 (75.85%)	1,474 (81.89%)	
High school or above	1910 (22.85%)	1,584 (24.15%)	326 (18.11%)	
Marital status				<0.001
Married	7,711 (92.26%)	6,100 (93.02%)	1,611 (89.50%)	
Other	647 (7.74%)	458 (6.98%)	189 (10.50%)	
Smoking				<0.001
Former	480 (5.74%)	367 (5.60%)	113 (6.28%)	
Never	5,717 (68.40%)	4,364 (66.54%)	1,353 (75.17%)	
Current	2,161 (25.86%)	1827 (27.86%)	334 (18.56%)	
Drinking				<0.001
Current alcohol user	1,542 (18.45%)	1,348 (20.56%)	194 (10.78%)	
Non-current alcohol user	6,816 (81.55%)	5,210 (79.44%)	1,606 (89.22%)	
Diabetes				<0.001
No	6,361 (76.11%)	5,095 (77.69%)	1,266 (70.33%)	
Yes	1997 (23.89%)	1,463 (22.31%)	534 (29.67%)	
Heart failure				<0.001
No	6,029 (72.13%)	5,209 (79.43%)	820 (45.56%)	
Yes	2,329 (27.87%)	1,349 (20.57%)	980 (54.44%)	
Lipid-lowering therapy				<0.001
No	7,033 (84.15%)	5,690 (86.76%)	1,343 (74.61%)	
Unknown	83 (0.99%)	0 (0.00%)	83 (4.61%)	
Yes	1,242 (14.86%)	868 (13.24%)	374 (20.78%)	

Continuous variables are presented as median (interquartile range) and categorical variables as *n* (%). Continuous variables were compared using the Wilcoxon rank-sum test and categorical variables using Pearson’s chi-square test. CKD, chronic kidney disease; BMI, body mass index; TG, triglycerides; TC, total cholesterol; HDL-C, high-density lipoprotein cholesterol; LDL-C, low-density lipoprotein cholesterol; SBP, systolic blood pressure; DBP, diastolic blood pressure; FPG, fasting plasma glucose; NLR, neutrophil-to-lymphocyte ratio; TyG, triglyceride–glucose index; NTI, NLR × TyG index.

**Table 2:** Baseline characteristics of hypertensive participants according to CKD status in NHANES.

Variable	Total (*N* = 9,182)	Non-CKD (*n* = 6,431)	CKD (*n* = 2,751)	*p* value
Age, years	57.00 (46.00–68.00)	55.00 (43.00–64.00)	69.00 (56.00–78.00)	<0.001
BMI, kg/m^2^	29.70 (25.92–34.50)	29.70 (26.00–34.35)	29.70 (25.75–35.00)	0.956
TG, mmol/L	1.35 (0.95–1.98)	1.33 (0.93–1.95)	1.42 (0.98–2.04)	0.006
TC, mmol/L	5.02 (4.32–5.77)	5.04 (4.40–5.79)	4.84 (4.16–5.64)	<0.001
HDL-C, mmol/L	1.29 (1.06–1.58)	1.29 (1.06–1.58)	1.27 (1.06–1.58)	0.116
LDL-C, mmol/L	2.95 (2.33–3.65)	2.97 (2.38–3.67)	2.79 (2.15–3.54)	<0.001
Serum creatinine, μmol/L	77.79 (64.53–89.28)	73.37 (62.76–85.75)	90.17 (70.72–114.92)	<0.001
Lymphocytes, ×10^9^/L	1.90 (1.50–2.30)	1.90 (1.60–2.30)	1.80 (1.40–2.30)	<0.001
Neutrophils, ×10^9^/L	3.90 (3.10–5.00)	3.80 (3.00–4.90)	4.20 (3.30–5.30)	<0.001
SBP, mmHg	131.00 (119.00–144.00)	129.50 (119.00–142.50)	138.00 (122.50–152.00)	<0.001
DBP, mmHg	74.00 (65.50–83.00)	75.00 (67.00–84.00)	70.00 (61.00–81.50)	<0.001
FPG, mg/dL	104.00 (96.00–116.00)	102.60 (95.00–113.00)	110.00 (99.00–130.00)	<0.001
TyG index	8.77 (8.36–9.20)	8.74 (8.34–9.16)	8.88 (8.45–9.33)	<0.001
NLR	2.08 (1.57–2.80)	2.00 (1.52–2.69)	2.32 (1.73–3.21)	<0.001
NTI	18.40 (13.73–24.88)	17.64 (13.30–23.70)	21.01 (15.48–28.43)	<0.001
Sex				<0.001
Female	4,661 (50.65%)	3,206 (49.04%)	1,455 (55.59%)	
Male	4,521 (49.35%)	3,225 (50.96%)	1,296 (44.41%)	
Race/ethnicity				0.334
Non-Hispanic White	4,242 (70.33%)	2,891 (70.62%)	1,351 (69.46%)	
Other race/ethnicity	4,940 (29.67%)	3,540 (29.38%)	1,400 (30.54%)	
Education				<0.001
Below high school	2,639 (19.22%)	1,698 (17.25%)	941 (25.23%)	
High school or above	6,535 (80.73%)	4,729 (82.71%)	1806 (74.68%)	
Unknown	8 (0.05%)	4 (0.04%)	4 (0.09%)	
Marital status				<0.001
Married	4,227 (49.26%)	3,077 (51.40%)	1,150 (42.73%)	
Other	4,955 (50.74%)	3,354 (48.60%)	1,601 (57.27%)	
Smoking				0.002
Former	2,886 (31.97%)	1925 (30.90%)	961 (35.25%)	
Never	4,609 (49.14%)	3,248 (49.27%)	1,361 (48.73%)	
Current	1,676 (18.81%)	1,250 (19.75%)	426 (15.96%)	
Unknown	11 (0.07%)	8 (0.08%)	3 (0.06%)	
Drinking				<0.001
Current alcohol user	5,341 (64.92%)	3,963 (68.63%)	1,378 (53.63%)	
Non-current alcohol user	3,841 (35.08%)	2,468 (31.37%)	1,373 (46.37%)	
Diabetes				<0.001
No	6,254 (73.23%)	4,784 (79.02%)	1,470 (55.56%)	
Unknown	48 (0.31%)	41 (0.36%)	7 (0.13%)	
Yes	2,880 (26.47%)	1,606 (20.62%)	1,274 (44.31%)	
Heart failure				<0.001
No	8,602 (94.83%)	6,195 (97.06%)	2,407 (88.02%)	
Unknown	44 (0.33%)	18 (0.19%)	26 (0.78%)	
Yes	536 (4.84%)	218 (2.75%)	318 (11.20%)	
Lipid-lowering therapy				<0.001
No	6,143 (67.73%)	4,586 (71.40%)	1,557 (56.54%)	
Unknown	9 (0.14%)	7 (0.14%)	2 (0.13%)	
Yes	3,030 (32.13%)	1838 (28.46%)	1,192 (43.33%)	

Continuous variables are presented as survey-weighted median (interquartile range); categorical variables are presented as unweighted n with survey-weighted percentages. Continuous variables were compared using survey-weighted Wilcoxon rank-sum tests and categorical variables using Rao–Scott chi-square tests. CKD, chronic kidney disease; NHANES, National Health and Nutrition Examination Survey; BMI, body mass index; TG, triglycerides; TC, total cholesterol; HDL-C, high-density lipoprotein cholesterol; LDL-C, low-density lipoprotein cholesterol; SBP, systolic blood pressure; DBP, diastolic blood pressure; FPG, fasting plasma glucose; NLR, neutrophil-to-lymphocyte ratio; TyG, triglyceride–glucose index; NTI, NLR × TyG index.

### 3.2 Association between NTI and prevalent CKD

Higher NTI was associated with greater odds of prevalent CKD in both cohorts (Table 3). When modeled continuously, each 1-unit increase in raw NTI was associated with an adjusted OR of 1.012 (95% CI 1.010–1.014; *p* < 0.001) in the hospital cohort and 1.021 (95% CI 1.013–1.029; *p* < 0.001) in NHANES. A graded association was also observed across cohort-specific NTI quartiles. In the fully adjusted Model 2, the ORs for Q2, Q3, and Q4 versus Q1 were 1.30 (95% CI 1.07–1.58), 1.88 (1.57–2.26), and 2.85 (2.38–3.40), respectively, in the hospital cohort. The corresponding ORs in NHANES were 1.28 (1.05–1.57), 1.52 (1.24–1.87), and 1.93 (1.56–2.38). P for trend was <0.001 in both populations. Adjustment attenuated the magnitude of the crude associations but did not materially alter their direction or graded pattern. For Q4 versus Q1, the OR decreased from 4.60 (95% CI 3.91–5.41) in the crude hospital model to 3.64 (3.06–4.32) in Model 1 and 2.85 (2.38–3.40) in Model 2. In NHANES, the corresponding ORs were 2.58 (2.15–3.09), 2.34 (1.91–2.86), and 1.93 (1.56–2.38). Thus, part of the crude association was accounted for by demographic and clinical covariates, while the association remained evident after full adjustment.

**Table 3:** Association between the NTI and prevalent CKD in sequential logistic regression models.

Characteristic	Crude model OR (95% CI)	*p*	Model 1 OR (95% CI)	*p*	Model 2 OR (95% CI)	*p*
Hospital population						
NTI, per 1-unit increase	1.020 (1.017–1.022)	<0.001	1.016 (1.014–1.019)	<0.001	1.012 (1.010–1.014)	<0.001
Q1	Reference		Reference		Reference	
Q2	1.42 (1.18–1.70)	<0.001	1.34 (1.11–1.62)	0.002	1.30 (1.07–1.58)	0.007
Q3	2.42 (2.04–2.86)	<0.001	2.10 (1.76–2.51)	<0.001	1.88 (1.57–2.26)	<0.001
Q4	4.60 (3.91–5.41)	<0.001	3.64 (3.06–4.32)	<0.001	2.85 (2.38–3.40)	<0.001
P for trend		<0.001		<0.001		<0.001
NHANES population						
NTI, per 1-unit increase	1.033 (1.026–1.040)	<0.001	1.027 (1.019–1.035)	<0.001	1.021 (1.013–1.029)	<0.001
Q1	Reference		Reference		Reference	
Q2	1.26 (1.05–1.52)	0.015	1.35 (1.11–1.63)	0.002	1.28 (1.05–1.57)	0.015
Q3	1.70 (1.40–2.07)	<0.001	1.76 (1.43–2.16)	<0.001	1.52 (1.24–1.87)	<0.001
Q4	2.58 (2.15–3.09)	<0.001	2.34 (1.91–2.86)	<0.001	1.93 (1.56–2.38)	<0.001
P for trend		<0.001		<0.001		<0.001

Crude model: unadjusted. Model 1: adjusted for age, sex, race/ethnicity, education, and marital status. Model 2: Model 1 plus smoking, drinking, BMI, systolic blood pressure, diabetes, and heart failure. NHANES models incorporate survey weights, strata, and primary sampling units. NTI quartiles were defined separately within each cohort using the final cohort-specific cutoffs. BMI, body mass index; CI, confidence interval; NHANES, National Health and Nutrition Examination Survey; NLR, neutrophil-to-lymphocyte ratio; NTI, NLR–TyG index; OR, odds ratio; TyG, triglyceride–glucose index.

### 3.3 Dose–response association

Restricted cubic spline analyses supported an overall association between NTI and prevalent CKD in both populations (all P-overall < 0.001; Figure 2). In the hospital cohort, evidence of nonlinearity remained after full adjustment (Model 2 P-nonlinear < 0.001), with the increase in odds becoming more pronounced at higher NTI values. In NHANES, evidence of nonlinearity was weaker and was not statistically significant after full adjustment (Model 2 P-nonlinear = 0.075); therefore, no statistically significant departure from linearity was detected.

**Figure 2:**
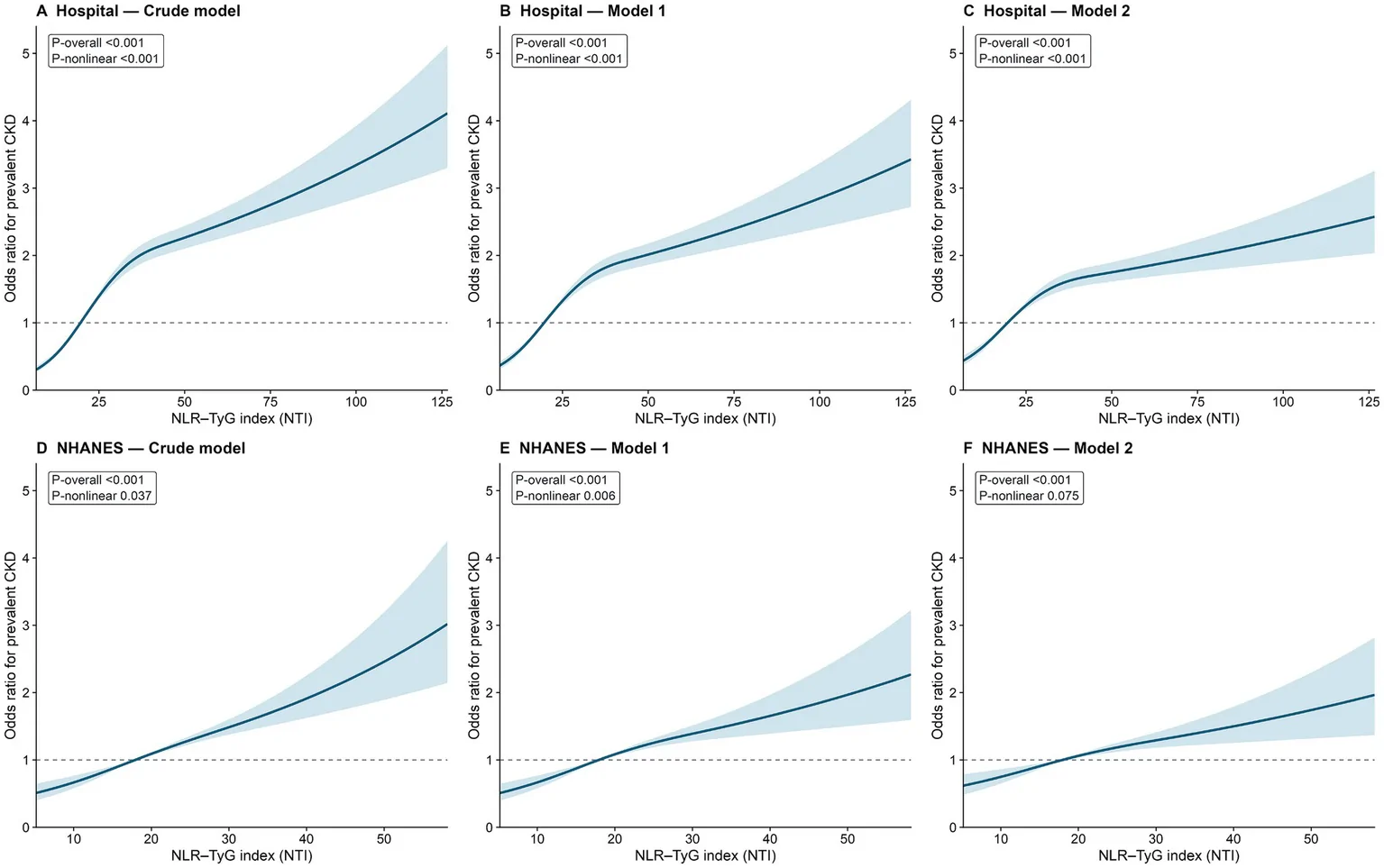
Restricted cubic spline associations of the NTI with prevalent chronic kidney disease. Panels **(A–C)** show the hospital cohort and panels **(D–F)** show NHANES, using crude, Model 1, and Model 2 specifications. Solid curves are odds ratios relative to the cohort-specific median NTI and shaded regions are 95% confidence intervals; dashed lines indicate OR = 1. Knots were placed at the 10th, 50th, and 90th percentiles of NTI. Model 1 adjusts for age, sex, race/ethnicity, education, and marital status; Model 2 additionally adjusts for smoking, drinking, BMI, systolic blood pressure, diabetes, and heart failure. P-overall was obtained from a joint spline-term test and P-nonlinear from the nonlinear spline component. NHANES models incorporated survey weights, strata, and primary sampling units. BMI, body mass index; NHANES, National Health and Nutrition Examination Survey; NLR, neutrophil-to-lymphocyte ratio; NTI, NLR–TyG index; OR, odds ratio; TyG, triglyceride–glucose index.

### 3.4 Sensitivity analysis

After excluding participants using lipid-lowering therapy and those with unknown medication status, 7,033 hospital participants (1,343 with CKD) and 6,143 NHANES participants (1,557 with CKD) remained. The continuous association between NTI and prevalent CKD persisted in the fully adjusted model, with an OR per 1-unit increase of 1.012 (95% CI 1.009–1.015; *p* < 0.001) in the hospital cohort and 1.026 (1.016–1.036; *p* < 0.001) in NHANES (Table 4). The quartile analyses were likewise consistent with the primary findings. Compared with Q1, the adjusted OR for Q4 was 2.79 (95% CI 2.28–3.42) in the hospital cohort and 2.05 (1.58–2.65) in NHANES, with P for trend <0.001 in both populations. The Q2 estimate was not statistically significant after full adjustment in either sensitivity cohort, whereas the Q3 and Q4 associations remained significant.

**Table 4:** Sensitivity analysis restricted to participants with known non-use of lipid-lowering therapy.

Characteristic	Crude model OR (95% CI)	*p*	Model 1 OR (95% CI)	*p*	Model 2 OR (95% CI)	*p*
Hospital population						
NTI, per 1-unit increase	1.020 (1.017–1.023)	<0.001	1.016 (1.014–1.019)	<0.001	1.012 (1.009–1.015)	<0.001
Q1	Reference		Reference		Reference	
Q2	1.34 (1.08–1.65)	0.006	1.25 (1.01–1.55)	0.042	1.17 (0.94–1.47)	0.155
Q3	2.40 (1.98–2.92)	<0.001	2.08 (1.70–2.55)	<0.001	1.79 (1.45–2.21)	<0.001
Q4	4.87 (4.05–5.86)	<0.001	3.80 (3.13–4.62)	<0.001	2.79 (2.28–3.42)	<0.001
P for trend		<0.001		<0.001		<0.001
NHANES population						
NTI, per 1-unit increase	1.036 (1.028–1.043)	<0.001	1.031 (1.022–1.040)	<0.001	1.026 (1.016–1.036)	<0.001
Q1	Reference		Reference		Reference	
Q2	1.10 (0.85–1.42)	0.472	1.22 (0.94–1.57)	0.132	1.18 (0.91–1.54)	0.212
Q3	1.54 (1.20–1.99)	0.001	1.64 (1.25–2.14)	<0.001	1.46 (1.12–1.90)	0.006
Q4	2.52 (2.02–3.16)	<0.001	2.42 (1.88–3.11)	<0.001	2.05 (1.58–2.65)	<0.001
P for trend		<0.001		<0.001		<0.001

Participants using lipid-lowering therapy and those with unknown medication status were excluded. Lipid-lowering therapy referred to use before admission in the hospital cohort and current use in NHANES. Crude model: unadjusted. Model 1: adjusted for age, sex, race/ethnicity, education, and marital status. Model 2: Model 1 plus smoking, drinking, BMI, systolic blood pressure, diabetes, and heart failure. NHANES models incorporate survey weights, strata, and primary sampling units. NTI quartiles were defined separately within each cohort using the final cohort-specific cutoffs and the same cutoffs were retained in the lipid-lowering sensitivity analysis. BMI, body mass index; CI, confidence interval; NHANES, National Health and Nutrition Examination Survey; NLR, neutrophil-to-lymphocyte ratio; NTI, NLR–TyG index; OR, odds ratio; TyG, triglyceride–glucose index.

### 3.5 Subgroup analyses

The positive association of continuous NTI with prevalent CKD was generally observed across the prespecified subgroups (Figure 3). In the hospital cohort, formal interaction tests indicated heterogeneity by sex (P for interaction = 0.030) and BMI (*p* = 0.043). The per-unit NTI association was somewhat stronger in women than in men (OR 1.015 vs. 1.011) and in participants with BMI ≥ 25 kg/m^2^ than in those with BMI < 25 kg/m^2^ (OR 1.018 vs. 1.011). Interactions with age, race/ethnicity, smoking, drinking, and diabetes were not statistically significant (all P for interaction > 0.05). In NHANES, interaction was observed for age (P for interaction = 0.023) and race/ethnicity (*p* = 0.005). The association was stronger among participants aged <60 years than among those aged ≥60 years (OR 1.037 vs. 1.010 per 1-unit NTI) and among participants categorized as other race/ethnicity than among non-Hispanic White participants (OR 1.036 vs. 1.015). Interactions with sex, smoking, drinking, diabetes, and BMI were not statistically significant. Because the interaction patterns differed between the hospital and NHANES populations, the subgroup findings were treated as exploratory.

**Figure 3:**
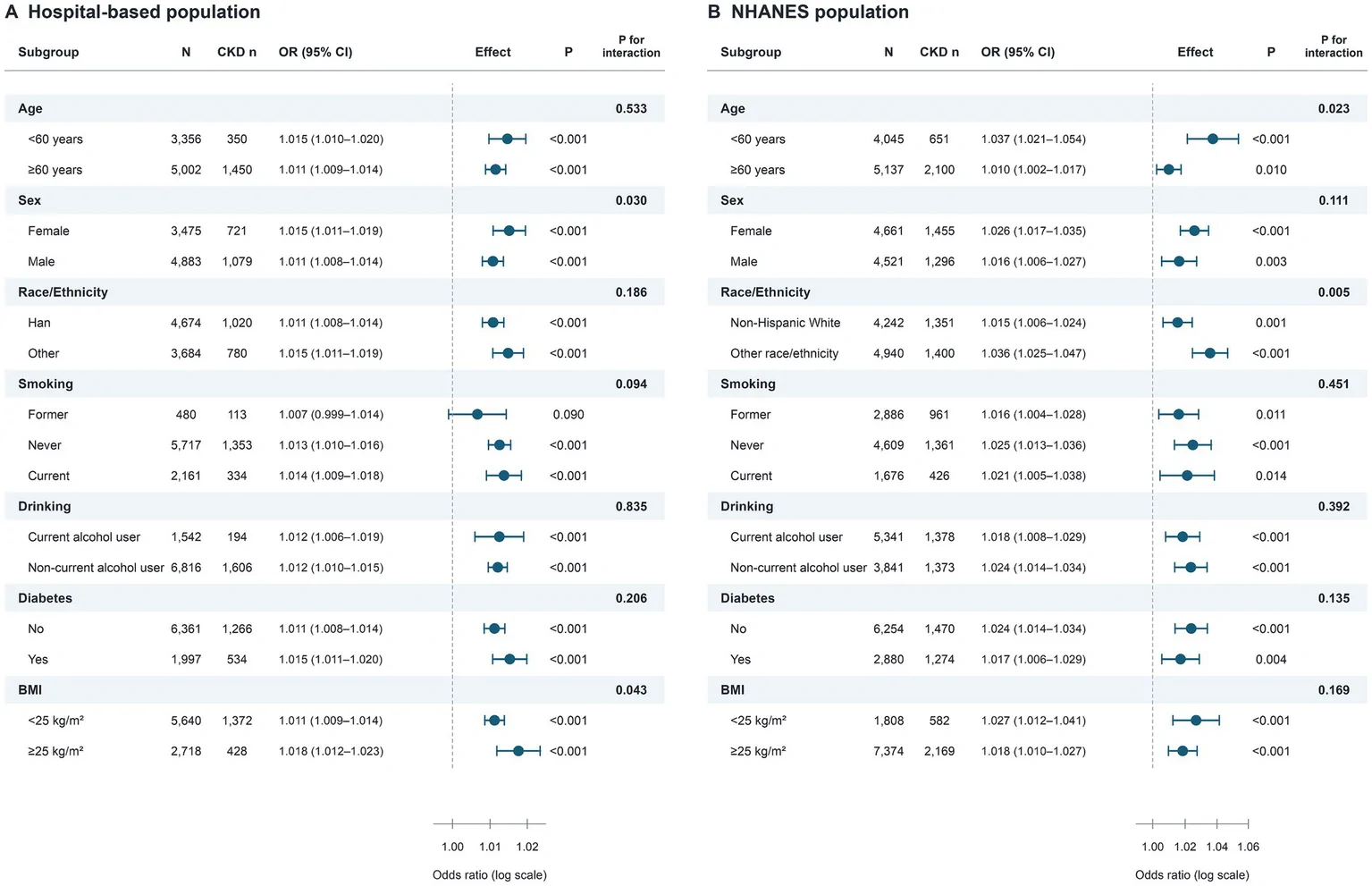
Associations of continuous NTI with prevalent chronic kidney disease across prespecified subgroups. **(A)** Hospital-based population and **(B)** NHANES population. Points show adjusted odds ratios per 1-unit increase in raw NTI and horizontal bars show 95% confidence intervals; the vertical dashed line indicates OR = 1. Stratum-specific models used the Model 2 adjustment set, omitting the stratification variable within each stratum. P for interaction was obtained from formal NTI-by-subgroup interaction testing using likelihood-ratio tests in the hospital cohort and survey-adjusted joint Wald *F* tests in NHANES. For NHANES smoking and diabetes analyses, the pre-existing Unknown category contributed to the joint interaction test but was omitted from the displayed subgroup rows. NHANES, National Health and Nutrition Examination Survey; NLR, neutrophil-to-lymphocyte ratio; NTI, NLR–TyG index; OR, odds ratio; TyG, triglyceride–glucose index.

### 3.6 Incremental discriminative performance and alternative parameterizations

Adding NTI to the basic clinical model produced modest improvements in discrimination (Table 5). In the hospital cohort, the C statistic increased from 0.769 (95% CI 0.756–0.781) for the basic model to 0.778 (0.766–0.790) after addition of NTI, corresponding to a change in C statistic of 0.009 (95% CI 0.007–0.014). The continuous NRI was 0.259 (95% CI 0.191–0.325) and the IDI was 0.0152 (0.0102–0.0216). The added NTI term improved model fit (likelihood-ratio χ^2^ = 117.98, *p* < 0.001). In NHANES, the C statistic increased from 0.772 (95% CI 0.763–0.783) to 0.778 (0.769–0.788), with a change in C statistic of 0.005 (95% CI 0.003–0.008), continuous NRI of 0.150 (0.099–0.199), and IDI of 0.0072 (0.0044–0.0111). The survey-adjusted model-comparison test was also significant (Wald *F* = 27.12, *p* < 0.001). The discrimination achieved by NTI was similar to that obtained when NLR and TyG were entered as separate terms: the C statistic for the latter model was 0.779 in both cohorts (Supplementary Table S2). TyG alone produced smaller gains, whereas NLR alone yielded discrimination close to that of NTI. In additional analyses, standardized log(NTI) remained positively associated with prevalent CKD (hospital OR per 1 standard deviation (SD) 1.491, 95% CI 1.406–1.581; NHANES OR 1.288, 95% CI 1.186–1.399; both *p* < 0.001). The formal NLR × TyG interaction was not significant in either the hospital cohort (*p* = 0.994) or NHANES (*p* = 0.686), indicating that the association of the composite did not depend on demonstrable multiplicative interaction between its components.

**Table 5:** Incremental discriminative performance of NLR, TyG, NTI, and separate NLR plus TyG terms for prevalent CKD.

Model	C statistic (95% CI)	ΔC (95% CI)	Continuous NRI (95% CI)	IDI (95% CI)	Model-comparison statistic	*p* value
Hospital population						
Basic model	0.769 (0.756–0.781)	Reference	Reference	Reference	Reference	
Basic + TyG	0.770 (0.757–0.782)	0.001 (0.000–0.003)	0.078 (0.025–0.139)	0.0013 (0.0001–0.0035)	LR χ^2^ = 11.47	0.001
Basic + NLR	0.778 (0.765–0.790)	0.009 (0.006–0.014)	0.256 (0.187–0.319)	0.0147 (0.0099–0.0211)	LR χ^2^ = 114.32	<0.001
Basic + NTI	0.778 (0.766–0.790)	0.009 (0.007–0.014)	0.259 (0.191–0.325)	0.0152 (0.0102–0.0216)	LR χ^2^ = 117.98	<0.001
Basic + NLR + TyG	0.779 (0.766–0.791)	0.010 (0.007–0.015)	0.254 (0.197–0.320)	0.0158 (0.0105–0.0225)	LR χ^2^ = 123.41	<0.001
NHANES population						
Basic model	0.772 (0.763–0.783)	Reference	Reference	Reference	Reference	
Basic + TyG	0.774 (0.766–0.785)	0.002 (0.000–0.004)	0.107 (0.049–0.161)	0.0022 (0.0004–0.0048)	Wald *F* = 9.00	0.003
Basic + NLR	0.777 (0.768–0.787)	0.004 (0.002–0.007)	0.150 (0.094–0.186)	0.0062 (0.0035–0.0100)	Wald *F* = 23.53	<0.001
Basic + NTI	0.778 (0.769–0.788)	0.005 (0.003–0.008)	0.150 (0.099–0.199)	0.0072 (0.0044–0.0111)	Wald *F* = 27.12	<0.001
Basic + NLR + TyG	0.779 (0.771–0.790)	0.007 (0.004–0.010)	0.162 (0.110–0.212)	0.0090 (0.0057–0.0136)	Wald *F* = 18.76	<0.001

The basic model included age, sex, race/ethnicity, education, marital status, smoking, drinking, BMI, systolic blood pressure, diabetes, and heart failure. ΔC, continuous NRI, and IDI are relative to the basic model; 95% confidence intervals were obtained from 250 bootstrap replicates. Hospital model comparisons used likelihood-ratio tests; NHANES comparisons used survey-adjusted Wald *F* tests. BMI, body mass index; IDI, integrated discrimination improvement; LR, likelihood ratio; NHANES, National Health and Nutrition Examination Survey; NLR, neutrophil-to-lymphocyte ratio; NRI, net reclassification improvement; NTI, NLR–TyG index; TyG, triglyceride–glucose index.

## 4 Discussion

### 4.1 Principal findings

In this cross-sectional study of adults with hypertension, higher NTI was independently associated with prevalent CKD in both a Chinese hospital-based cohort and the US NHANES population. In fully adjusted analyses, participants in the highest NTI quartile had higher odds of prevalent CKD than those in the lowest quartile in the hospital cohort (OR 2.85, 95% CI 2.38–3.40) and NHANES (OR 1.93, 95% CI 1.56–2.38). Restricted cubic spline analyses supported an overall dose–response association in both populations; the fully adjusted association was nonlinear in the hospital cohort, whereas no statistically significant evidence of nonlinearity was detected in NHANES. The association remained evident among participants with known non-use of lipid-lowering therapy. These findings are consistent with the broader cardiovascular–kidney–metabolic framework, in which hypertension, metabolic dysfunction, inflammation, and kidney disease represent interrelated components of systemic disease (3).

Adding NTI to the basic clinical model produced statistically detectable but modest gains in discrimination. The C statistic increased from 0.769 to 0.778 in the hospital cohort and from 0.772 to 0.778 in NHANES. Importantly, a model that included NLR and TyG as separate terms achieved discrimination very similar to that of the NTI model, and the formal NLR × TyG interaction was not significant in either cohort. Accordingly, the present findings support NTI as a parsimonious summary of concurrent inflammatory and metabolic burden rather than as evidence that the two components interact multiplicatively or that the product form is mathematically superior to modeling NLR and TyG separately.

### 4.2 Comparison with previous literature

The inflammatory component of NTI is supported by previous work linking neutrophil-predominant systemic inflammation with adverse kidney outcomes. NLR integrates relative neutrophil predominance and lymphocyte balance and has been associated with mortality, cardiovascular events, and progression to end-stage kidney disease among patients with CKD (26). In a Japanese cohort of patients with non-dialysis CKD, higher NLR was independently associated with poorer renal outcomes (27). In patients with diabetes, higher neutrophil counts have also been associated with subsequent CKD development (28), while analyses in diabetic kidney disease have related higher circulating neutrophil counts to adverse outcomes (29). These observations support the relevance of the inflammatory component of NTI, although they do not establish that elevated NLR precedes CKD in the present cross-sectional study.

A parallel body of evidence links the TyG index with kidney outcomes. TyG is primarily a surrogate marker of insulin resistance and glucose–lipid dysregulation rather than a kidney-specific biomarker. Nevertheless, higher TyG has been associated with incident CKD in prospective cohorts and meta-analyses (30, 31), as well as with CKD progression in patients with type 2 diabetes (32). Evidence in hypertensive populations is particularly relevant to the present study. In a Chinese hypertensive cohort, changes in TyG were associated with CKD progression (33), and a separate analysis in normal-weight hypertensive adults demonstrated a graded association between TyG and prevalent CKD (34). Sakoda et al. (35) also reported that higher TyG predicted subsequent CKD development across several blood-pressure phenotypes, including hypertensive groups. More recently, a large multicenter cohort of patients with CKD stages 3–4 found an independent association between baseline TyG and renal-function progression, with a nonlinear dose–response pattern (36).

A recent study by Söner et al. (37) further extends this literature. Söner et al. (37) reported that a higher TyG index was associated with contrast-induced nephropathy among patients with chronic total occlusion undergoing percutaneous coronary intervention. Although contrast-induced nephropathy and chronic CKD represent different clinical settings, this observation supports the broader concept that metabolic dysregulation may identify individuals with greater renal vulnerability. Complementary evidence from CKD cohorts has linked higher serum triglyceride concentrations with adverse renal outcomes and more rapid kidney-function decline (38–40).

Taken together, the available literature supports both inflammation and metabolic dysfunction as relevant domains in kidney disease. The present study extends this evidence by evaluating a simple composite in two hypertensive populations with different clinical and sampling characteristics. However, our data do not show that multiplying NLR by TyG creates a biologically synergistic marker. Rather, the similar discrimination obtained when NLR and TyG were entered separately suggests that the practical contribution of NTI lies mainly in providing a single, compact representation of two routinely available domains.

### 4.3 Potential biological mechanisms

Several biological pathways provide plausibility for the observed association between a higher inflammatory–metabolic burden and prevalent CKD. On the metabolic side, insulin resistance is closely linked to endothelial dysfunction, oxidative stress, hypertension, dyslipidemia, and altered energy metabolism in CKD (41). Abnormal lipid handling within the kidney may further contribute to glomerular and tubular injury. Experimental and translational studies indicate that dysregulated lipid uptake and lipid-droplet accumulation can promote oxidative stress, inflammation, and fibrotic signaling in renal tissue (42). Impaired mitochondrial fatty-acid oxidation may reduce adenosine triphosphate generation and favor lipid accumulation, tubular injury, and fibrosis (43), while renal lipotoxicity and mitochondrial dysfunction may reinforce one another (44). These mechanisms provide a biologically plausible context for the TyG component of NTI, but TyG should still be interpreted as a systemic metabolic surrogate rather than a direct measure of renal lipid deposition.

On the inflammatory side, neutrophil-predominant immune activation can contribute to endothelial and tissue injury through reactive oxygen species, proteases, cytokine signaling, and neutrophil extracellular traps (NETs). The NOD-like receptor family pyrin domain containing 3 inflammasome has been implicated in renal inflammation and fibrosis through interleukin-1β/interleukin-18 signaling and pyroptotic pathways (45, 46). Experimental studies further suggest that gasdermin D-dependent NET formation can promote renal fibrosis and macrophage-to-myofibroblast transition in obstructive nephropathy (47), while caspase-11/gasdermin D-dependent NET formation contributes to inflammatory and fibrotic injury in hyperuricemic nephropathy (48).

These mechanisms support the concept that inflammatory and metabolic abnormalities may coexist and jointly characterize a higher-risk biological state. Nevertheless, the present epidemiological data do not demonstrate a specific molecular interaction between the two pathways. The nonsignificant NLR × TyG interaction in both cohorts therefore argues for a cautious interpretation: NTI may summarize concurrent abnormalities in two relevant domains, but it should not be regarded as proof of multiplicative biological synergy.

### 4.4 Subgroup heterogeneity and cross-population consistency

The overall positive association between NTI and prevalent CKD was observed across most prespecified subgroups, but the interaction patterns differed between the two populations. In the hospital cohort, formal interaction was observed for sex and BMI, whereas in NHANES significant interaction was observed for age and race/ethnicity. In contrast, these interaction patterns were not reproduced across both cohorts. This lack of cross-population replication is important because subgroup interaction tests are particularly sensitive to differences in case mix, covariate distributions, sample size, and sampling design.

Accordingly, we do not interpret these subgroup findings as evidence of established biological effect modification. Instead, they should be regarded as exploratory and hypothesis-generating. The more consistent observation is that the direction of the NTI–CKD association was broadly preserved across clinically relevant strata in both populations. Future longitudinal studies specifically designed to evaluate effect modification may help determine whether any of the observed subgroup differences are reproducible.

### 4.5 Strengths and limitations

This study has several strengths. It evaluated the same composite exposure in two large and demographically distinct hypertensive populations, including a hospital-based Chinese cohort and a nationally representative US survey. The analyses incorporated cohort-specific survey methods for NHANES, evaluated NTI both continuously and by cohort-specific quartiles, characterized dose–response patterns with restricted cubic splines, and included sensitivity and formal interaction analyses. The comparison of NTI with NLR and TyG alone, as well as with NLR and TyG entered as separate terms, also provides a more balanced assessment of the composite rather than assuming superiority from its mathematical construction.

Several limitations should be considered. First, the study was observational and cross-sectional, and the hospital component was retrospective. Therefore, temporality cannot be established and reverse causation is possible. Residual or unmeasured confounding, selection bias, and information bias cannot be completely excluded despite multivariable adjustment and sensitivity analyses. Second, CKD ascertainment differed between the two data sources. NHANES incorporated both eGFR and albuminuria, whereas UACR was unavailable in the hospital cohort, where CKD was identified from eGFR and recorded CKD diagnosis. This difference may contribute to between-cohort heterogeneity and may introduce outcome misclassification. In addition, CKD classification based on single-occasion eGFR and UACR measurements may not establish chronicity in every participant, particularly in NHANES; the recorded CKD diagnosis available in the hospital cohort partly mitigates but does not eliminate this limitation. Third, NLR, triglycerides, and glucose were measured at a single time point and may not fully represent long-term inflammatory or metabolic status. In the inpatient cohort, acute illness or short-term physiological changes may have influenced NLR, glucose, triglycerides, and serum creatinine concurrently. Fourth, the hospital cohort consisted of inpatients with a greater clinical burden, whereas NHANES represents a community-based population; absolute NTI distributions and effect sizes should therefore not be assumed to transfer directly between settings. Complete-case exclusion for residual covariate missingness in NHANES may also have introduced additional selection bias if missingness was not completely random.

Fifth, multiple subgroup interaction tests were performed, and the statistically significant interaction patterns differed between cohorts. These findings should therefore be regarded as exploratory. Sixth, although NTI was prespecified as the product of NLR and TyG, the present study does not establish that multiplication is the optimal method for integrating the two components. The nonsignificant formal NLR × TyG interaction and the similar discrimination of the separate-component model reinforce this limitation. Finally, the discrimination analyses were cross-sectional and should not be interpreted as validation of prospective CKD prediction. Longitudinal studies with repeated biomarker measurements, harmonized CKD ascertainment, and external validation are needed before NTI can be considered for routine clinical risk assessment.

## 5 Conclusion

Higher NTI was independently associated with prevalent CKD among adults with hypertension in both a Chinese cardiology inpatient cohort and NHANES. The index provided a parsimonious summary of inflammatory-metabolic burden and modest incremental discrimination beyond conventional factors, with performance comparable to a model containing NLR and TyG as separate terms. These cross-sectional findings do not establish causality or prospective predictive utility and warrant longitudinal validation before NTI is considered for routine clinical risk assessment.

## Data Availability

The hospital cohort data are not publicly available because of institutional privacy policies and patient confidentiality restrictions, but de-identified data may be available from the corresponding author upon reasonable request, subject to institutional ethics approval and any required data-sharing agreements. NHANES data are publicly available from the US National Center for Health Statistics (https://www.cdc.gov/nchs/nhanes/). Analytic code may be made available from the corresponding author upon reasonable request.

## Glossary

AI: artificial intelligence
BMI: body mass index
CI: confidence interval
CKD: chronic kidney disease
CKD-EPI: Chronic Kidney Disease Epidemiology Collaboration
DBP: diastolic blood pressure
eGFR: estimated glomerular filtration rate
FPG: fasting plasma glucose
HbA1c: hemoglobin A1c
HDL-C: high-density lipoprotein cholesterol
ICD-10: International Classification of Diseases, Tenth Revision
IDI: integrated discrimination improvement
IQR: interquartile range
LDL-C: low-density lipoprotein cholesterol
LR: likelihood ratio
NETs: neutrophil extracellular traps
NHANES: National Health and Nutrition Examination Survey
NLR: neutrophil-to-lymphocyte ratio
NRI: net reclassification improvement
NTI: NLR–TyG index
OR: odds ratio
SBP: systolic blood pressure
SD: standard deviation
TC: total cholesterol
TG: triglycerides
TyG: triglyceride–glucose index
UACR: urine albumin-to-creatinine ratio

## References

[1] Wang L Xu X Zhang M Hu C Zhang X Li C . Prevalence of chronic kidney disease in China: results from the sixth China chronic disease and risk factor surveillance. JAMA Intern Med. (2023) 183:298–310. doi: 10.1001/jamainternmed.2022.6817, 36804760 PMC9941971

[2] Zeng X Zeng Q Zhou L Zhu H Luo J. Prevalence of chronic kidney disease among US adults with hypertension, 1999 to 2018. Hypertension. (2023) 80:2149–58. doi: 10.1161/hypertensionaha.123.21482, 37497635

[3] Ndumele CE Rangaswami J Chow SL Neeland IJ Tuttle KR Khan SS . Cardiovascular-kidney-metabolic health: a presidential advisory from the American Heart Association. Circulation. (2023) 148:1606–35. doi: 10.1161/cir.0000000000001184 37807924

[4] Buonacera A Stancanelli B Colaci M Malatino L. Neutrophil to lymphocyte ratio: an emerging marker of the relationships between the immune system and diseases. Int J Mol Sci. (2022) 23:3636. doi: 10.3390/ijms23073636, 35408994 PMC8998851

[5] Yu Y Lin Z Zhang W Jiang L Zhang C Ni X . The neutrophil-to-lymphocyte ratio and incident chronic kidney disease in a community-based cohort: a prospective study. Sci Rep. (2026) 16:29975. doi: 10.1038/s41598-026-58556-1 42380542

[6] Simental-Mendía LE Rodríguez-Morán M Guerrero-Romero F. The product of fasting glucose and triglycerides as surrogate for identifying insulin resistance in apparently healthy subjects. Metab Syndr Relat Disord. (2008) 6:299–304. doi: 10.1089/met.2008.0034, 19067533

[7] Sánchez-García A Rodríguez-Gutiérrez R Mancillas-Adame L González-Nava V Díaz González-Colmenero A Solis RC . Diagnostic accuracy of the triglyceride and glucose index for insulin resistance: a systematic review. Int J Endocrinol. (2020) 2020:1–7. doi: 10.1155/2020/4678526, 32256572 PMC7085845

[8] Lei L Liang H Qu Y Zhong Q Zhang Q Dai L . Association between triglyceride-glucose index and worsening renal function in the elderly. Front Nutr. (2022) 9:951564. doi: 10.3389/fnut.2022.951564, 36505264 PMC9730025

[9] Wang Y Gu C Chen B Qiu B Yu J. The association between triglyceride-glucose index and neutrophil-lymphocyte ratio and all-cause mortality in the general US population: NHANES 2001–2018. Front Endocrinol (Lausanne). (2024) 15:1513543. doi: 10.3389/fendo.2024.1513543, 39720246 PMC11666386

[10] Chen X Wang M Wu B Jiang W Yang Q Tang J . Reciprocal mediation of immuno-inflammation and insulin resistance in the risk of chronic kidney disease among patients with coronary artery disease: insights from neutrophil-to-lymphocyte ratio and triglyceride-glucose index. Front Immunol. (2026) 17:1937248. doi: 10.3389/fimmu.2026.1937248, 42780108 PMC13597357

[11] Lin Y Tian J Zeng Y Pan J Lin Z Hong F . Association and predictive value of the C-reactive protein-triglyceride-glucose index in chronic kidney disease. Sci Rep. (2026) (In press). doi: 10.1038/s41598-026-71235-5

[12] Akinbami LJ Chen T-C Davy O Ogden C Fink S Clark J . National Health and nutrition examination survey, 2017–march 2020 prepandemic file: sample design, estimation, and analytic guidelines. Vital Health Stat 2. (2022) 190:1–36. doi: 10.15620/cdc:115434 35593699

[13] Johnson CL Paulose-Ram R Ogden CL Carroll MD Kruszon-Moran D Dohrmann SM . National Health and nutrition examination survey: analytic guidelines, 1999–2010. Vital Health Stat 2. (2013) 161:1–24. 25090154

[14] National Center for Health Statistics. (2026). Ethics Review Board Approval: National Health and Nutrition Examination Survey: Centers for Disease Control and Prevention. Available online at: https://www.cdc.gov/nchs/nhanes/about/erb.html (Accessed October 13, 2026).

[15] Lin H Tang X Shen P Zhang D Wu J Zhang J . Using big data to improve cardiovascular care and outcomes in China: a protocol for the CHinese electronic health records research in Yinzhou (CHERRY) study. BMJ Open. (2018) 8:e019698. doi: 10.1136/bmjopen-2017-019698, 29440217 PMC5829949

[16] Pace R Peters T Rahme E Dasgupta K. Validity of health administrative database definitions for hypertension: a systematic review. Can J Cardiol. (2017) 33:1052–9. doi: 10.1016/j.cjca.2017.05.025, 28754391

[17] Crim MT Yoon SS Ortiz E Wall HK Schober S Gillespie C . National surveillance definitions for hypertension prevalence and control among adults. Circ Cardiovasc Qual Outcomes. (2012) 5:343–51. doi: 10.1161/circoutcomes.111.963439, 22550130 PMC3407684

[18] Levey AS Stevens LA Schmid CH Zhang Y Castro AF Feldman HI . A new equation to estimate glomerular filtration rate. Ann Intern Med. (2009) 150:604–12. doi: 10.7326/0003-4819-150-9-200905050-00006, 19414839 PMC2763564

[19] Kidney Disease: Improving Global Outcomes (KDIGO) CKD Work Group. KDIGO 2012 clinical practice guideline for the evaluation and management of chronic kidney disease. Kidney Int Suppl. (2013) 3:1–150.

[20] World Health Organization. (2026). Obesity and Overweight. Available online at: https://www.who.int/news-room/fact-sheets/detail/obesity-and-overweight (Accessed October 7, 2026).

[21] Chen G Khan N Walker R Quan H. Validating ICD coding algorithms for diabetes mellitus from administrative data. Diabetes Res Clin Pract. (2010) 89:189–95. doi: 10.1016/j.diabres.2010.03.007, 20363043

[22] McClure ST Schlechter H Oh S White K Wu B Pilla SJ . Dietary intake of adults with and without diabetes: results from NHANES 2013–2016. BMJ Open Diabetes Res Care. (2020) 8:e001681. doi: 10.1136/bmjdrc-2020-001681, 33099509 PMC7590352

[23] American Diabetes Association Professional Practice Committee. Diagnosis and classification of diabetes: standards of care in diabetes—2024. Diabetes Care. (2024) 47:S20–42. doi: 10.2337/dc24-s002 PMC10725812 38078589

[24] Desquilbet L Mariotti F. Dose-response analyses using restricted cubic spline functions in public health research. Stat Med. (2010) 29:1037–57. doi: 10.1002/sim.3841 20087875

[25] Pencina MJ D' Agostino RB Sr D' Agostino RB Jr Vasan RS. Evaluating the added predictive ability of a new marker: from area under the ROC curve to reclassification and beyond. Stat Med. (2008) 27:157–72. doi: 10.1002/sim.2929, 17569110

[26] Xu Y Chen Y Mai X Zhang M. Prognostic value of neutrophil-to-lymphocyte ratio for the clinical outcomes of chronic kidney diseases: an update systematic review and meta-analysis. BMC Nephrol. (2025) 26:419. doi: 10.1186/s12882-025-04363-1, 40721748 PMC12306010

[27] Yoshitomi R Nakayama M Sakoh T Fukui A Katafuchi E Seki M . High neutrophil/lymphocyte ratio is associated with poor renal outcomes in Japanese patients with chronic kidney disease. Ren Fail. (2019) 41:238–43. doi: 10.1080/0886022x.2019.1595645, 30942116 PMC6450582

[28] Zhang R Chen J Xiong Y Wang L Huang X Sun T . Increased neutrophil count is associated with the development of chronic kidney disease in patients with diabetes. J Diabetes. (2022) 14:442–54. doi: 10.1111/1753-0407.13292, 35789114 PMC9310049

[29] Xie R Bishai DM Lui DTW Lee PCH Yap DYH. Higher circulating neutrophil counts is associated with increased risk of all-cause mortality and cardiovascular disease in patients with diabetic kidney disease. Biomedicine. (2024) 12:1907. doi: 10.3390/biomedicines12081907, 39200371 PMC11352130

[30] Kunutsor SK Seidu S Kurl S Laukkanen JA. Baseline and usual triglyceride-glucose index and the risk of chronic kidney disease: a prospective cohort study. Geroscience. (2024) 46:3035–46. doi: 10.1007/s11357-023-01044-5, 38180700 PMC11009217

[31] Ren X Jiang M Han L Zheng X. Association between triglyceride-glucose index and chronic kidney disease: a cohort study and meta-analysis. Nutr Metab Cardiovasc Dis. (2023) 33:1121–8. doi: 10.1016/j.numecd.2023.03.026, 37088649

[32] Low S Pek S Moh A Ang K Khoo J Shao Y-M . Triglyceride-glucose index is prospectively associated with chronic kidney disease progression in type 2 diabetes – mediation by pigment epithelium-derived factor. Diab Vasc Dis Res. (2022) 19:14791641221113784. doi: 10.1177/14791641221113784, 35938490 PMC9364218

[33] Yu C Shi Y Wang T Zhu L Zhou W Bao H . Triglyceride–glucose index change and chronic kidney disease progression in a Chinese hypertensive population. Front Endocrinol (Lausanne). (2024) 15:1342408. doi: 10.3389/fendo.2024.1342408, 38405159 PMC10893760

[34] Yu C Zhou W Zhou X Zhu L Wang T Bao H . Association between triglyceride glucose index and chronic kidney disease in Normal-weight Chinese adults with hypertension. J Clin Hypertens (Greenwich). (2024) 26:1433–40. doi: 10.1111/jch.14913, 39400503 PMC11654844

[35] Sakoda T Akasaki Y Sasaki Y Kawasoe S Kubozono T Ikeda Y . Triglyceride-glucose index predicts future chronic kidney disease development in all populations, including normotensive and isolated diastolic hypertension. Hypertens Res. (2024) 47:149–56. doi: 10.1038/s41440-023-01507-4, 37989912

[36] Zhu F Mao H Gan W Zeng X Su L Chen W. Prognostic value of the triglyceride-glucose (TyG) index for renal function progression in patients with CKD stages 3–4. Front Nutr. (2026) 13:1744275. doi: 10.3389/fnut.2026.1744275, 42131235 PMC13161084

[37] Söner S Aktan A Kılıç R Güzel H Taştan E Okşul M . Association between the triglyceride-glucose index and contrast-induced nephropathy in chronic total occlusion patients undergoing percutaneous coronary intervention. BMC Cardiovasc Disord. (2025) 25:31. doi: 10.1186/s12872-025-04474-5, 39828693 PMC11744983

[38] Suh SH Oh TR Choi HS Kim CS Bae EH Oh K-H . Serum triglycerides level is independently associated with renal outcomes in patients with non-dialysis chronic kidney disease: results from KNOW-CKD study. Front Nutr. (2022) 9:1037618. doi: 10.3389/fnut.2022.1037618, 36505239 PMC9729769

[39] Pontremoli R Desideri G Arca M Temporelli PL Perrone V Dovizio M . Hypertriglyceridemia is associated with decline of estimated glomerular filtration rate and risk of end-stage kidney disease in a real-word Italian cohort: evidence from the TG-RENAL study. Eur J Intern Med. (2023) 111:90–6. doi: 10.1016/j.ejim.2023.02.019, 36906475

[40] Seki M Nakano T Tanaka S Kitamura H Hiyamuta H Ninomiya T . Associations between the serum triglyceride level and kidney outcome in patients with chronic kidney disease: the Fukuoka kidney disease registry study. J Atheroscler Thromb. (2024) 31:1556–70. doi: 10.5551/jat.64625, 38735756 PMC11537783

[41] Nakashima A Kato K Ohkido I Yokoo T. Role and treatment of insulin resistance in patients with chronic kidney disease: a review. Nutrients. (2021) 13:4349. doi: 10.3390/nu13124349, 34959901 PMC8707041

[42] Mitrofanova A Merscher S Fornoni A. Kidney lipid dysmetabolism and lipid droplet accumulation in chronic kidney disease. Nat Rev Nephrol. (2023) 19:629–45. doi: 10.1038/s41581-023-00741-w, 37500941 PMC12926870

[43] Jang H-S Noh MR Kim J Padanilam BJ. Defective mitochondrial fatty acid oxidation and lipotoxicity in kidney diseases. Front Med (Lausanne). (2020) 7:65. doi: 10.3389/fmed.2020.00065 PMC7080698 32226789

[44] Ge M Fontanesi F Merscher S Fornoni A. The vicious cycle of renal lipotoxicity and mitochondrial dysfunction. Front Physiol. (2020) 11:732. doi: 10.3389/fphys.2020.00732, 32733268 PMC7358947

[45] Huang G Zhang Y Zhang Y Ma Y. Chronic kidney disease and NLRP3 inflammasome: pathogenesis, development and targeted therapeutic strategies. Biochem Biophys Rep. (2023) 33:101417. doi: 10.1016/j.bbrep.2022.101417, 36620089 PMC9813680

[46] Ji Y Hua H Jia Z Zhang A Ding G. Therapy targeted to the NLRP3 Inflammasome in chronic kidney disease. Kidney Dis (Basel). (2024) 10:369–83. doi: 10.1159/000539496, 39430292 PMC11488838

[47] Wang Y Li Y Chen Z Yuan Y Su Q Ye K . GSDMD-dependent neutrophil extracellular traps promote macrophage-to-myofibroblast transition and renal fibrosis in obstructive nephropathy. Cell Death Dis. (2022) 13:693. doi: 10.1038/s41419-022-05138-4, 35941120 PMC9360039

[48] Wu F Chen C Lin G Wu C Xie J Lin K . Caspase-11/GSDMD contributes to the progression of hyperuricemic nephropathy by promoting NETs formation. Cell Mol Life Sci. (2024) 81:114. doi: 10.1007/s00018-024-05136-z, 38436813 PMC10912150

