# Supplementary material for: NLR–TyG index and prevalent chronic kidney disease in hypertension: a cross-population study
Source: Front Nutr. 2026 Oct 19;13:1917577. doi: 10.3389/fnut.2026.1917577 (PMC13724347; doi:10.3389/fnut.2026.1917577)
Supplement: Supplementary file 1 [file Supplementary_file_1.DOCX]

# Supplementary Material

**Supplementary Table S1.** Distribution and cohort-specific quartile cutoffs of the NLR–TyG index

| **Cohort** | **N** | **Mean ± SD** | **Median (IQR)** | **Range** | **Skewness** | **Shapiro–Wilk P** | **Q1/Q2** | **Q2/Q3** | **Q3/Q4** |
| --- | --- | --- | --- | --- | --- | --- | --- | --- | --- |
| Hospital | 8,358 | 26.36 ± 29.90 | 19.62 (14.00–28.81) | 2.51–1523.38 | 18.23 | <0.001 | 14.000 | 19.622 | 28.809 |
| NHANES | 9,182 | 20.15 ± 11.70 | 17.89 (13.12–24.36) | 0.09–257.13 | 4.83 | <0.001 | 13.118 | 17.893 | 24.360 |

Distribution summaries and quartile cutoffs are unweighted descriptive statistics. Shapiro–Wilk tests used a fixed-seed sample of up to 5,000 observations per cohort. IQR, interquartile range; SD, standard deviation.

**Supplementary Table S2.** Functional-form and component-model sensitivity analyses

| **Cohort** | **Analysis** | **Effect measure** | **Estimate** | **95% CI** | **P value** |
| --- | --- | --- | --- | --- | --- |
| Hospital | Standardized log(NTI) | OR per 1 SD | 1.491 | 1.406–1.581 | <0.001 |
| Hospital | NLR × TyG interaction | Interaction OR | 1.000 | 0.971–1.030 | 0.994 |
| Hospital | Basic + NTI | C statistic | 0.778 | 0.766–0.790 | — |
| Hospital | Basic + NLR + TyG | C statistic | 0.779 | 0.766–0.791 | — |
| NHANES | Standardized log(NTI) | OR per 1 SD | 1.288 | 1.186–1.399 | <0.001 |
| NHANES | NLR × TyG interaction | Interaction OR | 0.979 | 0.884–1.084 | 0.686 |
| NHANES | Basic + NTI | C statistic | 0.778 | 0.769–0.788 | — |
| NHANES | Basic + NLR + TyG | C statistic | 0.779 | 0.771–0.790 | — |

The interaction analysis tests statistical interaction between NLR and TyG and does not alter the prespecified NTI definition. Similar C statistics for NTI and separate NLR plus TyG terms support interpretation of NTI as a parsimonious composite rather than a statistically optimized interaction score.

**Supplementary Figure S1.** Distribution of the NTI in the hospital-based and NHANES cohorts. Histograms and empirical density curves display the observed NTI distribution; dashed vertical lines mark cohort-specific quartile cutoffs. Distribution summaries and quartile boundaries are descriptive and unweighted, whereas NHANES association models incorporate the complex survey design and fasting-subsample weights.
